# The apicoplast localized isocitrate dehydrogenase is needed for *de novo* fatty acid synthesis in the apicoplast of *Toxoplasma gondii*

**DOI:** 10.3389/fcimb.2025.1542122

**Published:** 2025-06-24

**Authors:** Ke Pan, Ao Zeng, Xiaodie Ruan, Xinyu Mo, Bang Shen, Junlong Zhao, Yanqin Zhou

**Affiliations:** Key Laboratory Preventive Veterinary of Hubei Province, College of Veterinary Medicine, Huazhong Agricultural University, Wuhan, Hubei, China

**Keywords:** *Toxoplasma gondii*, apicoplast, isocitrate dehydrogensase, metabolism, FAS 2

## Abstract

*Toxoplasma gondii* (*T. gondii*), an apicomplexan parasite, infects a wide range of warm-blooded animals and poses significant risks to human health. The fatty acid synthesis II (FASII) pathway in the apicoplast, which is the major source of fatty acids in parasites, is considered a potential drug target. The apicoplast also harbors some enzymes of central carbon metabolism, which are crucial for its survival, but their biological roles remain unclear. In this study, we focused on apicoplast-localized isocitrate dehydrogenase 1 (ICDH1) and deleted it using CRISPR-Cas9 technology. The *Δicdh1* mutant tachyzoites displayed markedly impaired growth kinetics, with further suppression under serum-deprived conditions. However, this deletion did not affect the viability or virulence of the *Δicdh1* mutant in mice. NADPH, a product of ICDH1-mediated decarboxylation of isocitrate, is an essential cofactor for fatty acid synthesis. Using ¹³C_6_ glucose as a metabolic carbon source, we showed that the mutant strains had reduced incorporation of glucose-derived carbons into medium-chain length fatty acids (C14:0 and C16:0). Additionally, the growth of the mutant was partially restored by supplementation with exogenous C14:0 and C16:0 fatty acids. These results indicate that ICDH1 deletion affects the FASII pathway and parasite growth. Consistent with previous studies, this study confirms that *T. gondii* has metabolic flexibility in the apicoplast that allows it to acquire fatty acids through various pathways.

## 1 Introduction

*T. gondii* is an intracellular parasitic protozoan with a complex life cycle involving intermediate hosts, such as humans and other warm-blooded animals, and definitive hosts, such as feline species. The intermediate hosts, humans, and other warm-blooded animals can be infected by ingesting undercooked or raw meat contaminated with tissue cysts or eating food or water contaminated with oocysts shed by felines. The parasite can undergo both asexual and sexual reproductive phases in its life cycle, producing tachyzoites, bradyzoites, and oocysts. Approximately one-third of the global population is chronically infected with *T. gondii*. The bradyzoites, an asexual reproduction phase, are the main form of chronic infection and can cause serious health problems for both humans and animals (Crawford et al., 2006). As an intracellular parasite, *T. gondii* can use glucose and glutamine as substrates to metabolize the compounds, providing energy and many important intermediates (Fleige et al., 2007; Blume et al., 2009; MacRae et al., 2012).

*T. gondii* and *Plasmodium* belong to the phylum *Apicomplexa*, both have mitochondrion obtained from an alpha-proteobacterium and a non-photosynthetic plastid called apicoplast from a cyanobacterium (Melo et al., 2000; Boucher and Yeh, 2019). A variety of biosynthetic pathways occur within the apicoplast of *T. gondii*, including the isoprene synthesis pathway, fatty acid *de novo* synthesis pathway, and heme *de novo* synthesis pathway, which are recognized as therapeutic targets for toxoplasmosis (Goodman and McFadden, 2007; Li et al., 2013; Bergmann et al., 2020). In addition, some enzymes in glycolysis and the tricarboxylic acid (TCA) cycle are also located in the apicoplast. The apicoplast-localized glycolytic enzymes triosephosphate isomerase 2(TPI2) and phosphor-glyceraldehyde dehydrogenase 2 (GADPH2) are critical for tachyzoite proliferation (Niu et al., 2022). Aconitase (ACO) and isocitrate dehydrogenase 1 (ICDH1) are enzymes mainly involved in the breakdown of citric acid to produce NADPH and α-ketoglutarate. ACO is a dual-targeting enzyme that localizes to both the apicoplast and mitochondria. When inhibited by sodium fluoroacetate, it can severely affect *T. gondii* growth and fatty acid synthesis (MacRae et al., 2012), but it is unclear which localization within the organelles is responsible for this serious outcome. While ICDH is a multifunctional enzyme that plays a critical role in energy metabolism, redox homeostasis, and cellular regulation. However, the significance of their localization to the apicoplast remains unclear. In 2008, Fleige et al. reported that the succinyl-CoA synthetase depletion mutant displayed a 30% reduction in growth rate, which could be restored by supplementation with 2 µM succinate in the tissue culture medium (Fleige et al., 2008).

These studies led us to suspect that the function of the tricarboxylic acid cycle within mitochondria is not necessary for tachyzoites; therefore, the reason for ACO inhibition leading to parasite death is that TCA enzymes localized in the apicoplast are more important for parasites. In this study, we analyzed the biological function of ICDH1. Our findings will contribute to further the understanding of plastid glucose metabolism enzymes and refinement of metabolic models.

## 2 Materials and methods

### 2.1 Experimental animals and materials

Six-week-old ICR mice were purchased from Hubei Provincial Center for Disease Control and Prevention. The experimental procedure was approved by the Scientific Ethics Committee of Huazhong Agricultural University (HZAUMO-2021-0182), following animal welfare guidelines and minimizing animal suffering. The type I RH strain and its mutant strain were cultured in human foreskin fibroblasts (HFF, obtained from ATCC, Maryland, USA) in DMEM supplemented with 2% FBS at 37°C with 5% CO2.

### 2.2 Plasmid construction

All primers used in this study are listed in **Supplementary Table S1**. Locus-specific CRISPR plasmids were generated by replacing the UPRT targeting guide RNA (gRNA) in pSAG1-Cas9-sgUPRT with corresponding gRNAs, using site-directed mutagenesis as described previously (Shen et al., 2014). The homologous template and drug screening tag for ICDH1 localization or gene deletion strain replacement were ligated to the pUC19 vector using the ClonExpress MultiS Cloning Kit (Vazyme Biotech, Nanjing, China), and the fragments required for the construction of the mutant strain were subsequently amplified. Primers used to amplify each fragment were listed in **Supplementary Table S1** and genomic DNA of RH was used as template for amplification of 5’- and 3’-homologous arms.

The plasmid pET-sumo-ICDH1 (**Supplementary Figure S2A**) truncation, which expressed a ICDH1 derived polypeptide, was constructed by cloning the corresponding ICDH1 fragment (from AA180 to AA 594) amplified from RH cDNA into the pET-sumo vector, through a one-step cloning kit (Vazyme Biotech, China).

### 2.3 Construction of mutant parasites

**Figure 1:**
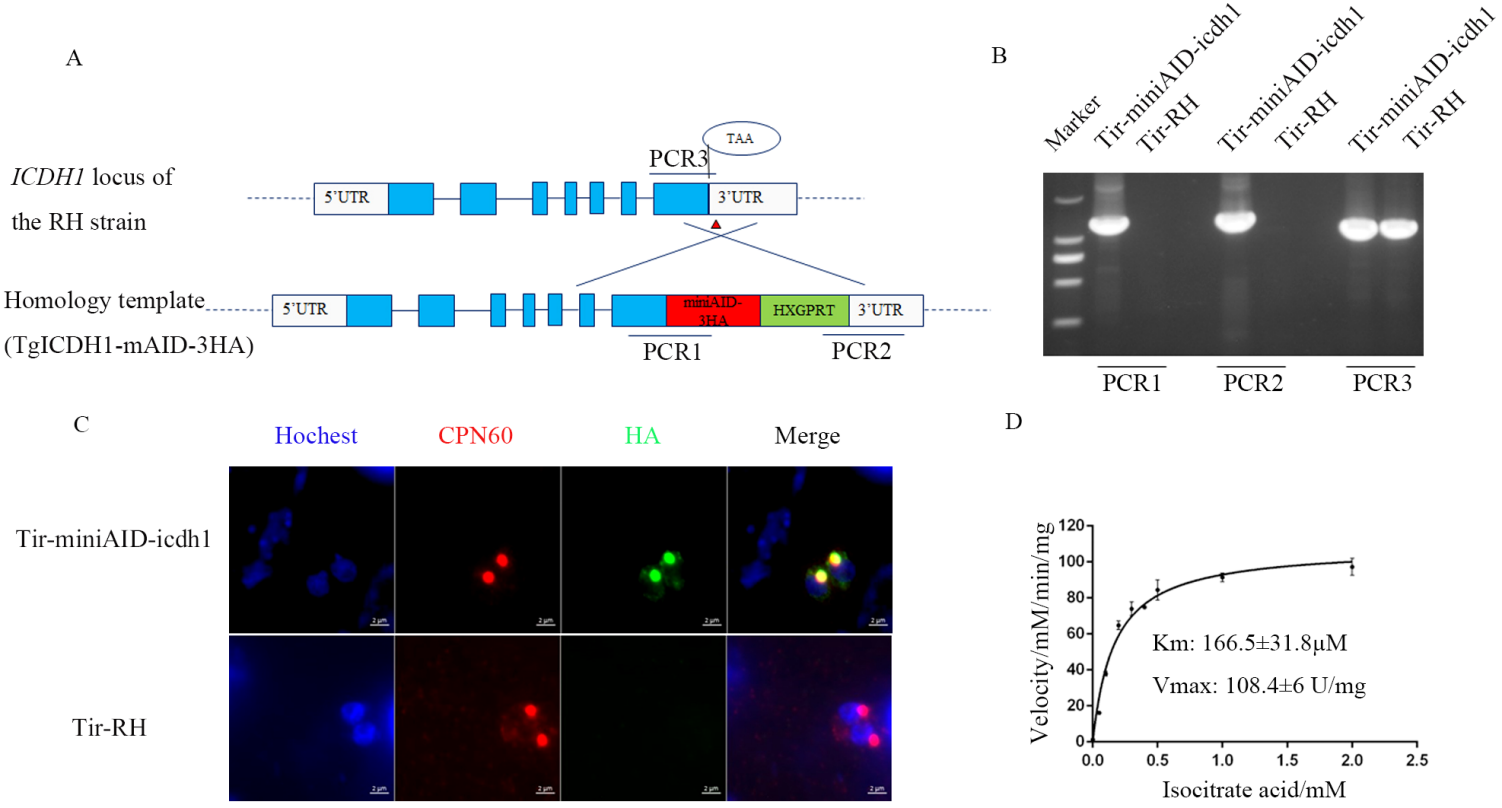
Apicoplast localization and kinetic characterization of *Tg*ICDH1. **(A)** Schematic diagram of the construction of a *Tg*ICDH1-localized strain. **(B)** PCR verification of the Tir-miniAID-icdh1 strain using primers PCR1, PCR2, and PCR3. **(C)** Indirect immunofluorescence assay of *Tg*ICDH1 localization in the Tir-miniAID-icdh1 strain. **(D)** *In vitro* enzyme kinetics of *Tg*ICDH1 using isocitrate as a substrate.

We used CRISPR-Cas9 technology to replace the target sequence with homologous fragments containing a selection marker, as shown in the **Figures 1A, B**. We screened the gene-edited strains with drugs such as 1 μM pyrimethamine or 30 μM chloramphenicol and obtained positive clones by restriction dilution and PCR verification. All primers and plasmids used in this study are listed in **Supplementary Table S1**.

Locus-specific CRISPR plasmids were generated by replacing the UPRT targeting guide RNA (gRNA) in pSAG1-Cas9-sgUPRT with corresponding gRNAs, using site-directed mutagenesis as described previously.

### 2.4 Analysis of parasites’ growth rates *in vivo*

To test the virulence of the mutant and RH strains, we purchased 7-week-old female ICR mice from Hubei Provincial Center of Disease Control and Prevention) were infected by intraperitoneal injection of the indicated strains (100 tachyzoites/mouse). The survival and symptoms of mice were monitored and recorded daily.

### 2.5 Analysis of parasites’ growth rates *in vitro*

We seeded *T. gondii* tachyzoites under optimal growth conditions (150 tachyzoites/well) in six-well plates with HFF monolayers, cultured them for 7 days, fixed and stained them with 4% paraformaldehyde and crystal violet. We also scanned the lysis plaques, and analyzed their sizes, as described in a previous report (Shen et al., 2014).

The replication rate analysis was performed according to (Xia et al., 2019). We inoculated 100 tachyzoites/well mutant or RH strains with an HFF monolayer on coverslips and incubated for 24 h. Then we incubated with rabbit-derived anti-TgALD polyclonal antibody (provided by Dr. David Sibley, University of Washington School of Medicine) at 1:2000 dilution for 20 min at room temperature, washed 5 times in PBS, secondary antibody Alexa Fluor 594-conjugated goat anti-rabbit IgG (Life Technologies, Camarillo, CA, USA) for 20 min at room temperature was added. After thorough washing, samples were observed under an Olympus BX53 microscope (Olympus Life Sciences, Tokyo, Japan) and the number of *T. gondii* in 100 vacuoles were counted.

For fatty acid supplementation experiments, we added 50 µM palmitic acid and/or cinnamic acid to the culture medium (purchased from Xi’an Kunchuang Technology Development Company).

### 2.6 Immunofluorescence assay

For immunofluorescence analysis, HFF monolayers on coverslips (in 24-well plates) were infected with 30μL of fresh parasite strains released from syringe-lysed HFF cells. After 45 minutes, non-invasive parasites were removed by washing with PBS. At 24 hours post-infection, the coverslips were fixed with 4% paraformaldehyde for 20 minutes and permeabilized with 0.1% Triton X-100 in PBS for 20 minutes. The primary antibody, diluted in PBS, was incubated at room temperature for 20 minutes. Following five washes with PBS, the secondary antibody (diluted in PBS) and nuclear stain (Hoechst) were co-incubated for 20 minutes at room temperature. The coverslips were then washed with PBS. Finally, the coverslips were mounted with Vectashield antifade mounting medium. Immunofluorescence images were captured using an Olympus BX53 microscope (Olympus Life Science, Japan) equipped with an Axiocam 503 monochrome camera (Carl Zeiss Inc., Germany) and ZEN 2 (blue version) software (Carl Zeiss Inc., Germany).

The primary antibodies were used as follows: HA monoclonal antibody (1:2000, Medical & biological laboratories Co., Ltd, Japan), rabbit anti-ALD (1:2000,provided by Dr. David Sibley at Washington University School of Medicine), mouse anti-Toxoplasma (1:1000,prepared in the laboratory),Alexa Fluor 488 conjugated Goat anti-Mouse IgG (1:2000 for IFA), Alexa Fluor 594 conjugated Goat anti-Mouse IgG (1:2000), Alexa Fluor 488 conjugated Goat anti-Rabbit IgG (1:2000) and Alexa Fluor 594 conjugated Goat anti-Rabbit IgG (1:2000) (Fisher Scientific. USA).

### 2.7 Prokaryotic expression and enzyme activity assay

We constructed a pET-sumo-ICDH1 plasmid containing the fragment of interest, transformed it into a BL21 strain, and induced expression of the protein of interest with 0.5 mM IPTG at 16°C for 16 h. We purified the protein with His-tag resin and measured its enzyme activity using the Solarbio@ ICDHm kit. We varied the concentration of isocitrate and NADP+, the sample concentration, as well as the buffer ion concentration, and monitored the production of NADPH by spectrophotometer at 340 nm. We calculated the reaction rate at each substrate concentration and fitted the Michaelis-Menten curve using GraphPad Prim6.0 software to obtain Km and Vmax values (NADPH has a molar extinction coefficient of 6.22 mm^-1^at 340 nm) (34). For the detection of ICDH enzyme activity using the kit previously mentioned, the 10uL purified sample was added to the 190uL working solution, and the initial absorbance value A1 was recorded at 340 nm wavelength for 20 seconds. The 96-well UV plate containing the above mixture was then placed in a 37°C water bath, the bottom of the plate was dried after 2 minutes of reaction, and the absorbance value A2 was quickly recorded in the microplate reader for 2 minutes and 20 seconds. The enzyme activity was calculated according to the provided formula ICDHc(U/mg prot)= [V(total reaction volume)×ΔA÷(ϵ×d)×10^9^]÷(W÷V(tested protein volume×Cpr)÷T=1608×ΔA/Cpr. ΔA=A2-A1, ϵ (Molar extinction coefficient of NADPH) = 6.22×10^3^L/mol/cm; Cpr: concentration of enzyme. d, 96-well plate optical path (0.6 cm) V, volume; T, reaction time; W: Sample quality.

### 2.8 Fatty acid labeling analysis

To assess the effect of *Tg*ICDH1 deletion on fatty acid synthesis, wild-type and Δ*icdh1* mutant parasites were cultured in medium supplemented with 8 mM ¹³C_6_ glucose for 48 hours. Infected host cells were then lysed using a syringe, and the intracellular parasites were purified using a 3-µm filter. The parasite pellets were processed for fatty acid extraction and derivatization following a previously described method (Bian et al., 2017). The collected intracellular tachyzoites was added 500 μL water and the mixture was transferred to a new glass tube and 500 μL methanol and 1 mL chloroform were added. The mixture was vortexed for 1 min and stood for 30 min. After centrifugation at 14000 g for 15 min at 4°C, 800 μL chloroform layer was evaporated to dryness. Afterwards, 500 μL of 0.5M KOH (75% ethanol) was added and held at 80°C for 60min. Then, 100 μL formate and 600 μL n-hexane were added. A layer of 500 μL n-hexane was evaporated to dryness. The dry residues were mixed with 10 μL HoBt (in DMSO), 20 μL cholamine (in DMSO with 200 mM TEA) and 10 μL HATU (in DMSO) and the resulting mixture was incubated at room temperature for 5 min. A 60 μL acetonitrile was added and followed by centrifuging at 14000 g for 15 min at 4°C prior to UHPLC-HRMS analysis. The obtained data were naturally isotope corrected according to the previous reports (Millard et al., 2019).

The injection volume was 0.5 μL and the flow rate was 0.3 mL/min. The column temperature was 40°C. The mobile phases consisted of water (phase A) and acetonitrile (phase B), both with 0.1% formate. A linear gradient elution was performed with the following program: 0 min, 10%B; 4 min, 30% B; 8min, 45% B; 11 min, 50%B; 14 min, 70%B; 15 min, 100%B and held to 18 min; 18.1 min, 10%B and held to 20 min. Spray voltage was set to 4000 V. Capillary and Probe Heater Temperature were separately 320°C and 320°C. Sheath gas flow rate was 35 (Arb, arbitrary unit), and Aux gas flow rate was 10 (Arb). S-Lens RF Level was 50 (Arb). The full scan was operated at a high-resolution of 70000 FWHM (m/z=200) at a range of 180–600 m/z with AGC Target setting at 3×10^6^.

### 2.9 Experimental data analysis

All experiments in this study were repeated more than 3 times at different times. Data analysis was performed using Student’s tests and/or one-way analysis of variance (ANOVA). Enzyme viability, virulence analysis and plotting were performed using Graph Prism 6.0.

## 3 Results

### 3.1 *Tg*ICDH1 has dehydrogenase activity in the apicoplast

Previous studies have identified two isocitrate dehydrogenases (ICDHs) in *T. gondii*: *Tg*ICDH1 (TGGT1_266760) and *Tg*ICDH2 (TGGT1_313140). Pino et al. (2007) demonstrated that *Tg*ICDH1 is localized to the apicoplast through ectopic expression of epitope-tagged genes. To further confirm the subcellular localization of *Tg*ICDH1, we tagged the endogenous *Tg*ICDH1 gene with a mAID-HA tag in the TIR1 strain and analyzed the corresponding transgenic lines using immunofluorescence microscopy (**Figure 1A**). The tagging of this gene was successfully performed on the *RHΔku80* strain by PCRs verification (**Figure 1B**). Immunofluorescent staining showed that *Tg*ICDH1 was expressed in the apicoplast, as indicated by colocalization with the organelle marker *Tg*CPN60 (**Figure 1C**).

ICDH is a key enzyme in the tricarboxylic acid cycle, mainly catalyzing the dehydrogenation of isocitrate. To test whether *Tg*ICDH1 contributes to NADPH for the occurrence of other reactions in the apicoplast, we performed enzymatic activity assays. We also evaluated the sequence alignment with the ICDHs of other species to determine the number of amino acids in the leader peptide (**Supplementary Figure S1**). The recombinant protein, purified from *E. coli* and approximately 59.7 kDa (**Supplementary Figure S2B**), was measured for its enzymatic activity using an NADPH-coupled assay. The results showed that it had dehydrogenase activity (4637 U/mg) with a Km value of 160.7 µM for isocitrate (**Figure 1D**).

### 3.2 *Tg*ICDH1 is required for the rapid growth of parasites

**Figure 2:**
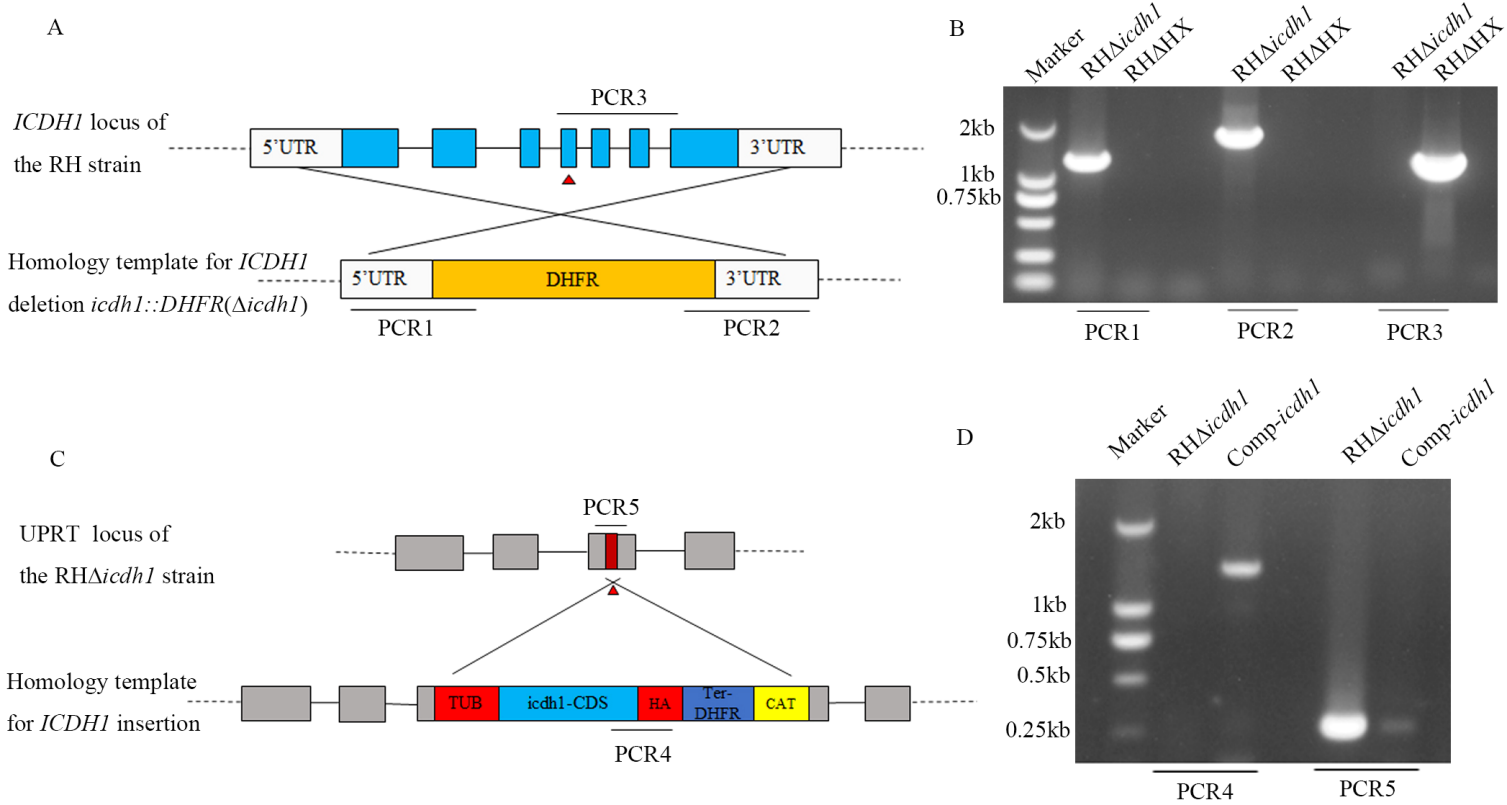
Generation and validation of the *Tg*ICDH1 mutant strain. **(A)** Schematic diagram of the strategy for constructing RHΔ*icdh1* strains based on homologous recombination and CRISPR Cas9. **(B)** PCR confirmation of the knockout of the endogenous *Tg*ICDH1 gene in RHΔ*HX*. **(C)** Schematic diagram of the strategy for constructing Comp-*icdh1* strain by ectopic complementation. **(D)** PCR verification of the Comp-*icdh1* strain.

To investigate the biological role of *Tg*ICDH1 during parasite growth, we knocked out the gene by using a CRISPR/Cas9-mediated homologous gene replacement strategy. *Tg*ICDH1 was completely replaced by the pyrimethamine-resistant cassette *DHFR* (dihydrofolate reductase), and the mutants were verified by PCRs in the strain RH (**Figures 2A, B**). To further confirm the effect of *Tg*ICDH1 deletion on the parasite, we complemented the mutant strain by co-transfecting a complementing plasmid containing *Tg*ICDH1 homologous fragment to the UPRT locus of *RHΔicdh1 strain*. The complemented clones were confirmed by PCRs. (**Figures 2C, D**).

**Figure 3:**
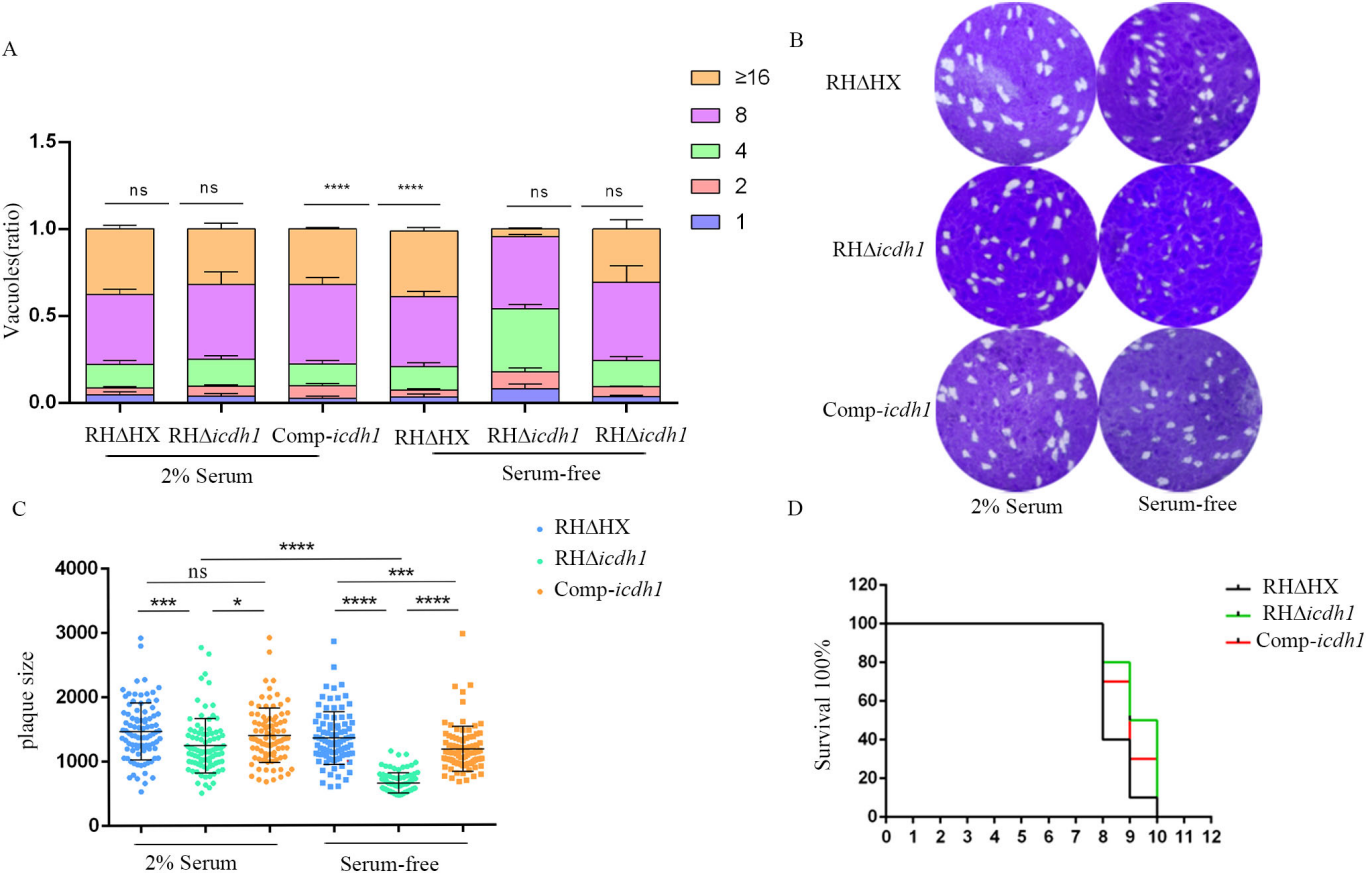
TgICDH1 deficiency impairs T. gondii growth. **(A)** Analysis of parasite replication rates by counting the number of parasites per vacuole (1, 2, 4, 8, 16, or more) showed that RHDicdh1 grew slower than wild-type and Comp-icdh1 strains. (ns,P>0.05;****, P<0.0001; one-way ANOVA with Bonferronis post-test). **(B)** Plaque assay of RHDicdh1 growth rate compared to wildtype and Comp-icdh1 strains. **(C)** Analysis of the plaque size formed by different strains. Data are presented as mean ± SD of three independent experiments. (ns, P>0.05; *,P ≤ 0.05; P<***, P< 0.001; ****, P<0.0001; one-way ANOVA with Bonferronis post-test). **(D)** Survival curves of mice infected with different strains (Each mouse is infected with 100/Tg through the intraperitoneal injection).

The lysis cycle, driven by tachyzoites of the parasite’s life stage, involves several biological processes, including invasion, replication, and egress. Parasite replication was impaired in Δ*icdh1* mutant strains under culture conditions with 2% serum, resulting in a significant decrease in the number of parasites per vacuole with an internal *T. gondii* number greater than 16 (**Figure 3A**). The plaque experiment showed that the plaques of the ICDH1-KO strain were different from those expressing ICDH1 (RH*ΔHX* and comp-*icdh1*) (**Figure 3B**). The plaque size also showed that the replication ability of the missing strain was weakened (**Figure 3C**). Growth defects were more severe in the RH*Δicdh1* mutant strains in serum-free DMEM culture (**Figures 3A-C**). These results suggest that *Tg*ICDH1 is important for optimal growth of *T. gondii in vitro*, but not essential for parasite survival. Phenotypic analysis of ICDH1 complementary strains revealed some recovery of the observed *Δicdh1* mutant deficiency (**Figure 3**). In addition, we found that ICDH1 is required for robust growth of the parasites in serum-free medium. We found that serum affected the replication of the mutant strain, likely due to the addition of fatty acids and other essential substances absent in the serum free condition, and the effect of fatty acids on the replication of *T. gondii* was also shown in previous studies (Krishnan et al., 2020; Liang et al., 2020; Chen et al., 2025).

To confirm whether the growth defect observed in the Δ*icdh1* mutant was a direct consequence of ICDH1 inactivation, we complemented the Δ*icdh1* strain with C-terminally HA-tagged *Tg*ICDH1 expressed from the UPRT locus (**Figure 2C**). Screening PCRs confirmed the desired integration of the complementing construct (**Figure 2D**). To confirm whether the growth defects observed in the Δ*icdh1* mutant are a direct result of ICDH1 inactivation, we supplemented the Δpdh-e1α strain with C-terminal HA-tagged *Tg*ICDH1 expressed at the UPRT locus (**Figure 2C**). Screening PCR confirmed the desired integration of complementary constructs (**Figure 2D**). Phenotypic analysis of ICDH1 complementary strains revealed some recovery of the observed Δ*icdh1* mutant deficiency (**Figure 3**). In addition, depending on the effect of the absence of ICDH1 in culture conditions containing 2% serum, we found that ICDH1 is required for robust growth of the parasites in serum-free medium.

### 3.3 *Tg*ICDH1 is dispensable for the parasite virulence

Despite impaired intracellular replication, our results assert that the parasite can survive without *Tg*ICDH1. To evaluate the impact of *Tg*ICDH1 on the vivo growth of *T. gondii*, we infected ICR mice with wild-type or mutant strain and monitored the survival of infected animals. We found that the two strains had very similar survival curves, indicating that the loss of *Tg*ICDH1 did not affect the acute virulence of the parasite (**Figure 3D**).

### 3.4 Fatty acid synthesis is reduced in the *Δicdh1* mutant

**Figure 4:**
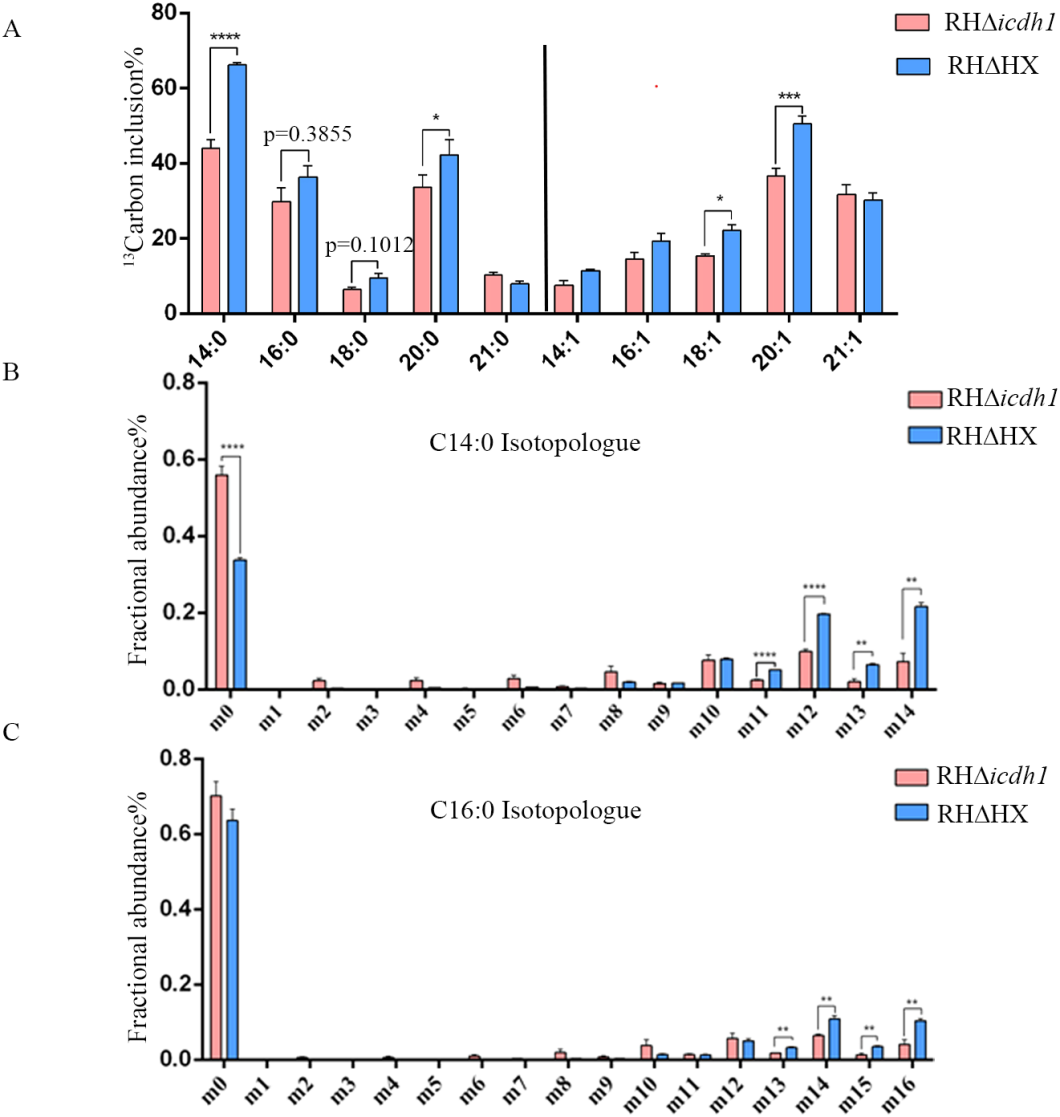
TgICDH1 deficiency decreases fatty acid synthesis. Intracellular wild-type and mutant parasites were labeled with 8 mM 13C6 glucose for 48 hand fatty acids derived from tachyzoites and were extracted and analyzed by GC-MS. **(A)** Percentage of each 13C6-labeled fatty acid relative to its total amount. **(B, C)** Amount of each 13C6-labeled isomer in myristic acid and palmitic acid (as a percentage of the labeled total). Data are presented as mean ± SE of four experiments (*,P< 0.05; **,P< 0.01; ***,P<0.001; ****,P<0.0001; Student’s t test).

To determine whether the phenotypic defects of the mutant strains were due to reduced NADPH levels in the apicoplast, thereby affecting fatty acid synthesis via the FASII pathway, we cultured parasites in glucose-free medium supplemented with ¹³C_6_ glucose for 48 hours. Subsequently, we analyzed the labeled fatty acid products using GC-MS. The results showed that the tracer incorporation of ^13^C_6_ glucose-derived carbons into myristic acid (C14:0) and palmitic acid (C16:0) was decreased in the mutant strain compared to the parent strain, with the most significant difference in the incorporation of myristic acid (**Figure 4A**). Isotopic analysis of myristic acid revealed that the wild-type strain had a high proportion of m12, m13, and m14 isotopes (corresponding to 12, 13, and 14 carbon label ^13^C), while the Δ*icdh1* mutant strain had mostly m0 isotopes (no ^13^C label) (**Figure 4B**). A similar pattern was observed for palmitic acid (C16:0) (**Figure 4C**). In the study of Ramakrishnan et al., myristic acid and palmitic acid were reported to be produced by the FASII pathway (Ramakrishnan et al., 2012), so it is believed that the deletion of ICDH1 reduces the synthesis of glucose-derived carbon into medium-chain fatty acids.

**Figure 5:**
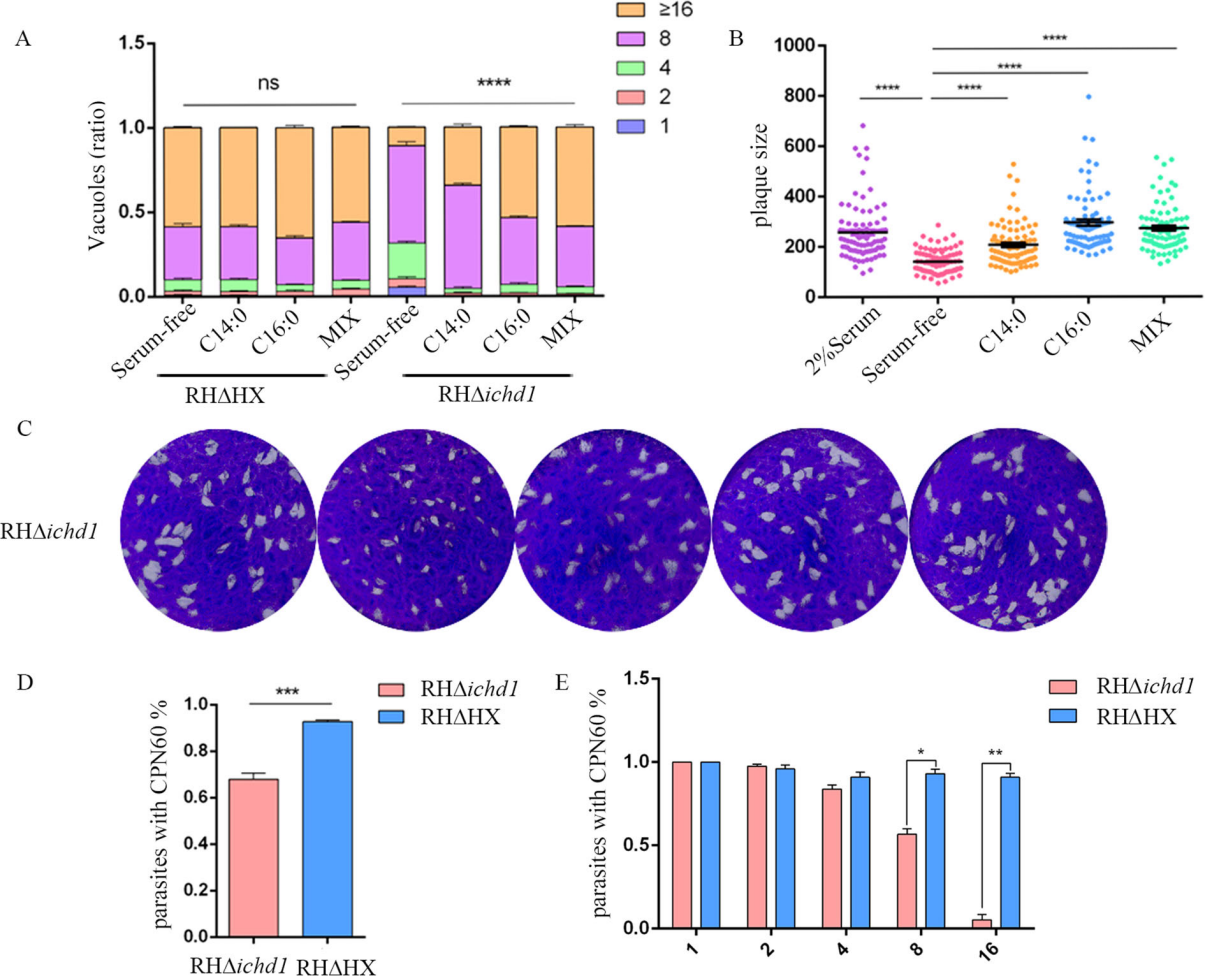
Exogenous fatty acids rescue the growth defect of the Dicdh1 mutant. **(A)** Replication rates of wild-type and mutant parasites with or without 100 mM myristic acid (C14:0), 100 mM palmitic acid (C16:0), or a mixture of fatty acids (Mix, 50 mm myristic acid + 50 mm palmitic acid). (ns, P>0.05;****, P< 0.0001; one-way ANOVA with Bonferroni’s post-test). **(B)** Plaque sizes obtained from C [arbitrary units (a.u.)]. (****, P< 0.0001; one-way ANOVA with Bonferroni’s post-test). **(C)** Plaque analysis in the presence (2% Serum) or absence (Serum-free) of 2% Serum, 100 mM myristic acid (C14:0), 100 mM palmitic acid (C16:0), or a mixture of fatty acids (Mix, 50 mm myristic acid + 50 mm palmitic acid). **(D)** Ratio of intracellular parasites’ apicoplast. (***, P<0.001; one-way ANOVA with Bonferronis post-test). **(E)** Correlation between the number of T. gondii in the vacuole and the loss of the apicoplast. (*, P < 0.05; **, P< 0.01; Student’s t test).

*T. gondii* can scavenge fatty acids from host cells. These fatty acids cross the parasitophorous vacuole membrane (PVM) to be available to the parasites (Fleige et al.,2008; Pino et al., 2007; Xu et al., 2021). To verify whether this mechanism can compensate for the loss of *Tg*ICDH1, we supplemented two fatty acids that were added exogenously and perform plaque and replication assays in the absence or presence of 100 μm myristic acid, palmitic acid, or a mixture of both (50 μm each). As expected, the supplementation indeed improved the growth of the Δ*icdh1* mutant, as shown by restoration in plaque size and replication assays with either fatty acid in **Figures 5A-C**.

### 3.5 The *Δicdh1* mutants show apicoplast loss

It has been reported that the disruption of the FASII pathway leads to serious defects in parasite development (Xu et al., 2021). Therefore, we stained the parasites with rabbit-derived polyclonal antibody against the apicoplast marker protein *Tg*CPN60 to detect whether the Δ*icdh1* mutants cause apicoplast loss in the *T. gondii*. Nearly all parasites harbored a plastid in the parental strain, as indicated by the bright *Tg*CPN60 signal. However, in *Δicdh1* mutants, some parasites had no apicoplast (**Figure 5D**; **Supplementary Figure S3**) when the number of tachyzoites in the parasitophorous vacuole was 8 or more and almost 32% of the tachyzoites lost their apicoplast (**Figure 5E**). These results suggest that *Tg*ICDH1 is important for maintaining the apicoplast and providing reducing power for lipid synthesis.

## 4 Discussion

*T. gondii* expresses two isocitrate dehydrogenases (ICDHs) with distinct gene codes: ICDH1 and ICDH2, which localize to the apicoplast and mitochondrion, respectively (Pino et al., 2007). In this study, we explore the function and biological significance of apicoplast-localized ICDH1. We showed that it had dehydrogenase activity and produced NADPH. Deletion of *Tg*ICDH1 led to a reduced replication rate of tachyzoites, a phenotype that was more pronounced in the absence of serum (**Figure 3**). Additionally, we observed the incorporation of [¹³C_6_] glucose derivatives into C14:0 (myristic acid) and C16:0 (palmitic acid), suggesting that NADPH generated by ICDH1 is crucial for maintaining *de novo* fatty acid synthesis in the apicoplast. However, this effect can be compensated by other NADPH-producing pathways within the apicoplast.

The entire growth process of *T. gondii* encompasses extracellular movement, invasion, and multiple cycles of intracellular replication and egress. Both the extracellular motility and invasion phases, as well as the egress phase, demand substantial energy production to enable total motility (Buscaglia et al., 2007; Ginger et al., 2010). During the intracellular stage, tachyzoites replicate rapidly and synthesize numerous macromolecules, such as fatty acids, which are crucial for constructing membrane structures. Thus, the supply of fatty acids is critical for intracellular parasite replication and can be obtained through various pathways. For instance, during the rapid proliferation phase of tachyzoites, exogenous acetate can be utilized to elongate fatty acid chains, thereby maintaining their growth (Ramakrishnan et al., 2012, 2015; Nitzsche et al., 2017).The apicoplast FASII pathway can *de novo* synthesize medium-chain fatty acids such as myristic acid and palmitic acid, and the endoplasmic reticulum fatty acid elongation pathway can produce long-chain unsaturated fatty acids required for growth (Dubois et al., 2018).The FASII pathway, as a *de novo* fatty acid synthesis pathway, is considered an important drug target for toxoplasmosis in *T. gondii*. However, similar to previous studies, the FASII pathway disrupted by ICDH1 deletion can be rescued by supplementation with exogenous fatty acids (Krishnan et al., 2020; Liang et al., 2020; Chen et al., 2025). This demonstrates the diversity and plasticity of the fatty acid acquisition routes of *T. gondii* (Pernas et al., 2018).

The TCA cycle provides substrates for numerous metabolic pathways in many organisms, maintaining mitochondrial ETC homeostasis and ATP production, and ICDH is often considered a key enzyme role in the TCA cycle in mitochondria. However, the deletion of succinyl CoA synthetase in *T. gondii* (Fleige et al., 2008), the intra-mitochondrial pyruvate carrier *Tg*MPC (Lyu et al., 2023), and intramitochondrial pyruvate dehydrogenase *Tg*BCKDH (Oppenheim et al., 2014)did not affect the *in vitro* growth of *T. gondii*, despite reducing the flux of glucose into the TCA cycle. Similarly, the successful deletion of six TCA cycling enzymes in malaria parasites (Ke et al., 2015) was not lethal, suggesting that the TCA cycle is not essential for the growth of the tachyzoite stage of the apicomplexan parasite. Cryptosporidium protozoa lack most of TCA cycle-related enzymes (Abrahamsen et al., 2004). However, the inhibition of ACO by high doses of NaFAC resulted in severely stunted growth of *T. gondii* and was thought to be due to the disruption of the TCA cycles (MacRae et al., 2012). Malate: quinone oxidoreductase(MQO), as a membrane protein that participates in ETC, TCA cycle, fumarate cycle, and other pathways, can inhibit the growth of *Plasmodium falciparum* when inhibited by ferulenol (Niikura et al., 2017; Hartuti et al., 2018), and *T. gondii* also has this enzyme and can perform the same function, which is also inhibited ferulenol (Acharjee et al., 2021). The significance of the TCA cycle in *Plasmodium* metabolism is still controversial (Jacot et al., 2016; Nitzsche et al., 2016; Kloehn et al., 2020), but it is considered important for the sexual replication stage in the mosquito vector (MacRae et al., 2013). However, due to the limitations of studying of the sexual phase of *T. gondii*, we cannot evaluate the role of the TCA cycle during this period. We also tried to delete ICDH2 in the mitochondrion of *T. gondii* after successfully deleting ICDH1 in the apicoplast, but we failed with multiple deletion strategies. This suggests that ICDH in the mitochondria and the TCA cycle are important for *T. gondii* growth.

## Data Availability

The original contributions presented in the study are included in the article/**Supplementary Material**. Further inquiries can be directed to the corresponding author/s.
