# Supplementary material for: The apicoplast localized isocitrate dehydrogenase is needed for de novo fatty acid synthesis in the apicoplast of Toxoplasma gondii
Source: Front Cell Infect Microbiol. 2025 Jun 24;15:1542122. doi: 10.3389/fcimb.2025.1542122 (PMC12234471; doi:10.3389/fcimb.2025.1542122)
Supplement: Supplementary file 1 [file DataSheet1.pdf]

Supplementary Material

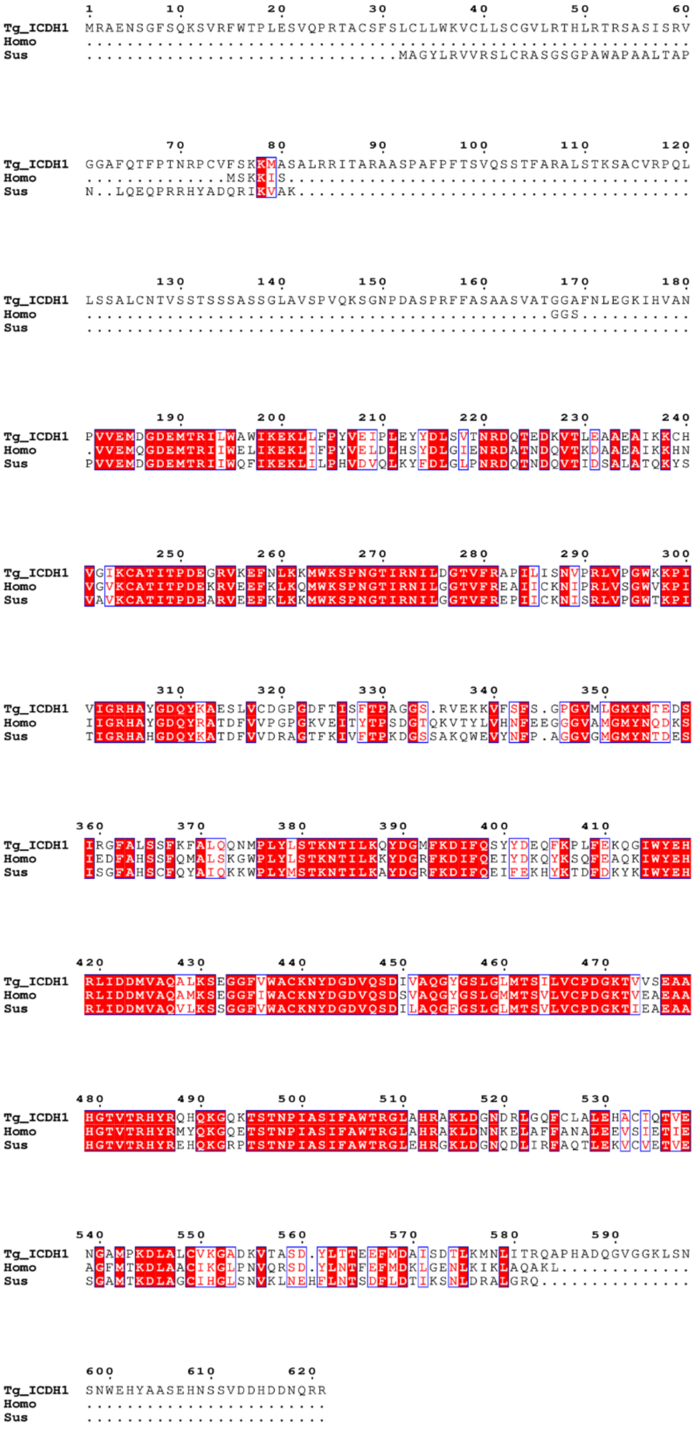

Figure S1. Alignment of amino acid sequences of isocitrate dehydrogenase (ICDH) in *T.gondii* and selected species(1).

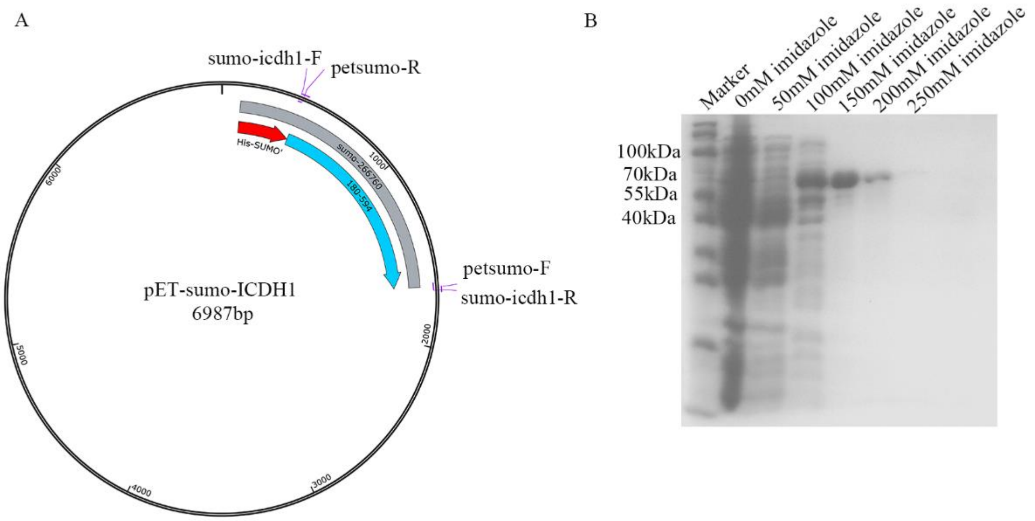

Figure S2. In vitro prokaryotic expression and purification of isocitrate dehydrogenase (ICDH1). (A) Map of pET-sumo-ICDH1 expression vector. (B) SDS-PAGE assessing the purity and concentration of purified recombinant ICDH1 proteins.

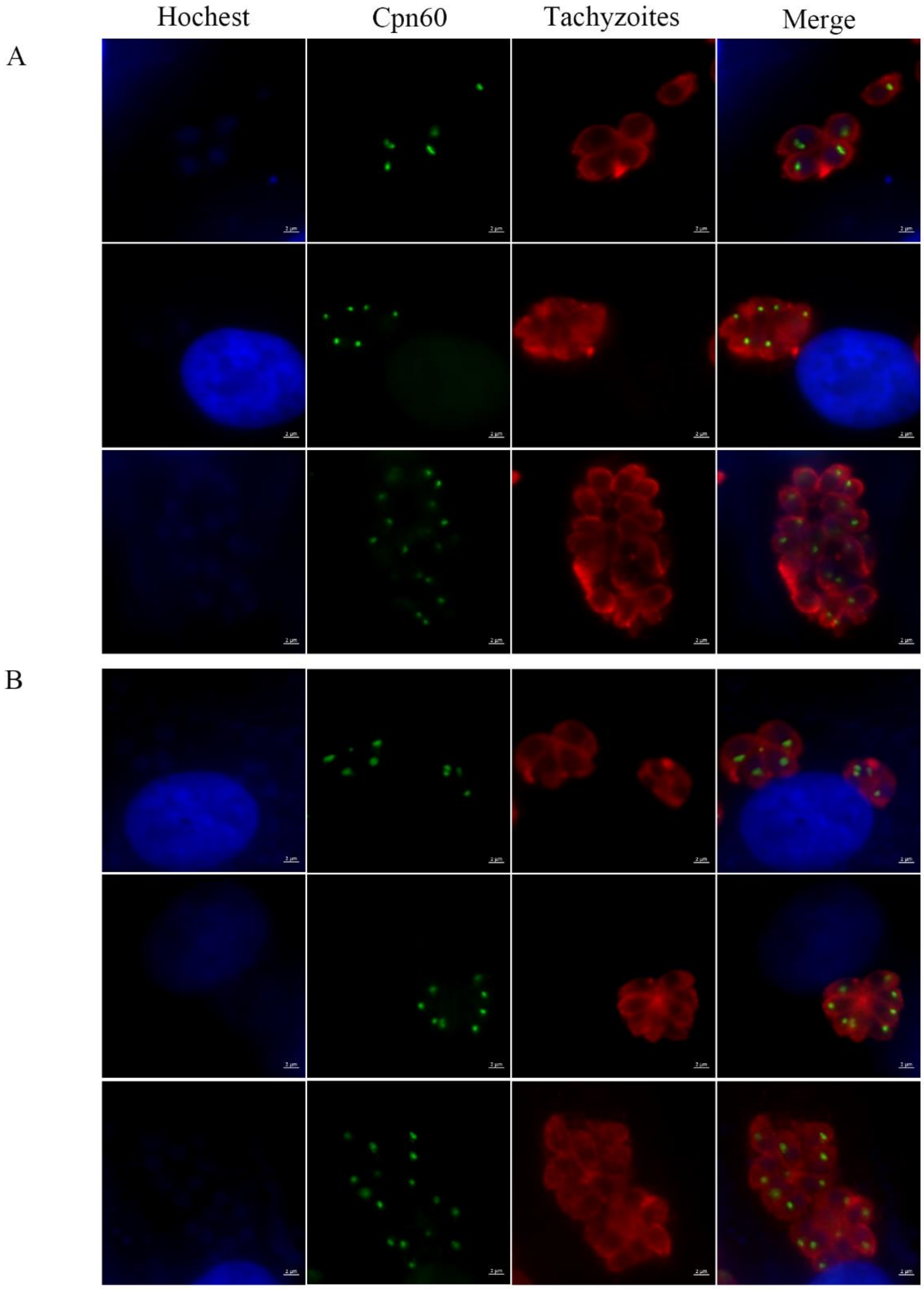

Figure S3. *Tg*ICDH1 tachyzoites' apicoplasts loss. (A)The number of apicoplasts in the RHΔ*icdh1* with only 4, 8 and 16 tachyzoites in the vacuole. (B)The number of apicoplasts in the RHΔHX with only 4, 8 and 16 tachyzoites in the vacuole.

Table S1: Primers used in this study

Primer	Sequence（5’-3’）	Used for
miniAID- icdh1 -5’-F	GAGAGTGCACCATATGGCAAGTATCAAAGGATTTGCACACTC	Plasmid for icdh1 localization
miniAID- icdh1-5’-R	GCTCACCATCCTAGGCGCTTTACTTATGATCTGTCGTTCC	Plasmid for icdh1 localization
miniAID- icdh1-3’-F	CTGAATGGCGAATGGGGGGCATGTTGTTCATCTGACTCG	Plasmid for icdh1 localization
miniAID- icdh1-3’-R	GGATCCCCGGGTACCCCCAGCTGATGCATATTCTAGCATC	Plasmid for icdh1 localization
miniAID- icdh1-gRNA-F	GGAATGGGGGAGCTTTCTTGGTTTTAGAGCTAGAAATAGC	Construction of gRNA
gRNA-icdh1-miniAID-F	GGAATGGGGGAGCTTTCTTGGTTTTAGAGCTAGAAATAGC	CRISPR plasmid vector for TgICDH1 -localization gRNA sequence
gRNA-icdh1-miniAID-R	AACTTGACATCCCCATTTAC	CRISPR plasmid vector for TgICDH1 -localization gRNA sequence
JD-miniAID-PCR1-F	GTGATGAGCTTCATCCCTGCTGACAAG	Verification of icdh1 localization
JD-miniAID-PCR1-R	GAACATCGTAAGGATACGCATAATCGGGC	Verification of icdh1 localization

JD-miniAID-PCR2-F	GGAGCTCCAATTCGCCCTATAGTGAG	Verification of icdh1 localization
JD-miniAID-PCR2-R	GCTGTCAACGAAAGTGGTGCTGTGG	Verification of icdh1 localization
JD-miniAID-PCR3-F	GTGATGAGCTTCATCCCTGCTGACAAG	Verification of icdh1 localization
JD-miniAID-PCR3-R	CAAGAAAGCTCCCCCATTCCAG	Verification of icdh1 localization
icdh1-miniAID-F	GCAAGTATCAAAGGATTTGCACACTC	Amplification of pUC19-miniAID
icdh1-miniAID-R	CCCAGCTGATGCATATTCTAGCATC	Amplification of pUC19-miniAID
QC-icdh1-5H-F	GTTGTAAAACGACGGCCAGTCCTGGGATGCCGTACCTATG	Plasmid for icdh1 knockout
QC- icdh1-5H-R	GATGTCTTCTGCGCGGGTTGGGTATGTGCAAAACAAATTA	Plasmid for icdh1 knockout
QC- icdh1-3H-F	GCCACAAGTTCAGCGTGTCCACTTGTGCCATCAGACGTAG	Plasmid for icdh1 knockout
QC- icdh1-3H-R	GCTATGACCATGATTACGCCTCGATAAGATGGCTCTGTGC	Plasmid for icdh1 knockout
QC- icdh1-F	ACTTGTGCCATCAGACGTAGCTTGC	Plasmid for icdh1 knockout

QC- icdh1-R	TCGATAAGATGGCTCTGTGCACCG	Plasmid for icdh1 knockout
gRNA-icdh1-F	AGTCGCCAAATGCAACGATCGTTTTAG AGCTAGAAATAGC	CRISPR plasmid vector for TgICDH1 gRNA sequence
gRNA-icdh1-R	AACTTGACATCCCCATTTAC	CRISPR plasmid vector for TgICDH1 gRNA sequence
JD- icdh1-RCR1-F	CAGACACTACGCTGTAAGTTG	Verification of icdh1 knockout
JD- icdh1-RCR1-R	GATTTGTGAGGACGACTCAC	Verification of icdh1 knockout
JD- icdh1-RCR2-F	CACGACAGCAGACAACTTTC	Verification of icdh1 knockout
JD- icdh1-RCR2-R	CACACGTGTGTTAACTTAGGC	Verification of icdh1 knockout
JD- icdh1-RCR3-F	CCTCGTGAAGCACATTGACCTAG	Verification of icdh1 knockout
JD- icdh1-RCR3-R	GAACATCTCCGTCATAATTCTTGCACG	Verification of icdh1 knockout
comp- icdh1-F	CTAAAATGGTGAGCAAGATGCGGGCA GAGAATTCCGGTTTTTCG	Plasmid for complementation
comp- icdh1-R	GGAACATCGTAAGGATATCGTCTCTGG TTGTCATCATGGTC	Plasmid for icdh1 complementation
PD- comp- icdh1-F	ATGCGGGCAGAGAATTCCGGTTTTTC	Amplification of the 5' homologous arm of icdh1 based on CAT tag

PD- comp- icdh1-R	GGCATAATCTGGAACATCGTAAGG	Amplification of the 5' homologous arm of icdh1 based on CAT tag
JD-comp- icdh1-PCR4-F	CCATCCTCAGCTTCCTGCAAG	Verification of icdh1 complementation
JD-comp- icdh1-PCR4-R	GGCATAATCTGGAACATCGTAAGG	Verification of icdh1 complementation
JD-comp- icdh1-PCR5-F	GGGTGCCTACGTTCTTCTAC	Verification of icdh1 complementation
JD-comp- icdh1-PCR5-R	GTCCCCAGGTAGCGAGAACG	Verification of icdh1 complementation
pET-sumo- icdh1-F	CGCGAACAGATTGGAGGTTTCCAGCGAATCAAGGTGGACCAGC	Plasmid for icdh1 prokaryotic expression
pET-sumo- icdh1-R	CGCGAACAGATTGGAGGTGGGGGGCATTCAACTTGGAG	Plasmid for icdh1 prokaryotic expression
pET-sumo-vector-F	ACTAGAGGATCCGAATTCGAGCTCC	Amplification of SUMO linearized vector
pET-sumo-vector-R	ACCTCCAATCTGTTCGCGGTGAG	Amplification of SUMO linearized vector

1. Robert X, Gouet P. 2014. Deciphering key features in protein structures with the new ENDscript server. Nucleic Acids Res 42:W320-4.
